# Treating cryptosporidiosis: A review on drug discovery strategies

**DOI:** 10.1016/j.ijpddr.2024.100542

**Published:** 2024-04-20

**Authors:** Anne-Charlotte Lenière, Alexis Vlandas, Jérôme Follet

**Affiliations:** University of Lille, CNRS, Centrale Lille, Junia, Université Polytechnique Hauts de France, UMR 8520, IEMN Institut d’Electronique de Microélectronique et de Nanotechnologie, F, 59000, Lille, France

**Keywords:** *Cryptosporidium*, Drug screening, Drug repurposing, Metabolic pathways

## Abstract

Despite several decades of research on therapeutics, cryptosporidiosis remains a major concern for human and animal health. Even though this field of research to assess antiparasitic drug activity is highly active and competitive, only one molecule is authorized to be used in humans. However, this molecule was not efficacious in immunocompromised people and the lack of animal therapeutics remains a cause of concern. Indeed, the therapeutic arsenal needs to be developed for both humans and animals. Our work aims to clarify research strategies that historically were diffuse and poorly directed. This paper reviews *in vitro* and *in vivo* methodologies to assess the activity of future therapeutic compounds by screening drug libraries or through drug repurposing. It focuses on High Throughput Screening methodologies (HTS) and discusses the lack of knowledge of target mechanisms. In addition, an overview of several specific metabolic pathways and enzymatic activities used as targets against *Cryptosporidium* is provided. These metabolic processes include glycolytic pathways, fatty acid production, kinase activities, tRNA elaboration, nucleotide synthesis, gene expression and mRNA maturation. As a conclusion, we highlight emerging future strategies for screening natural compounds and assessing drug resistance issues.

## Highlights

• Up to now, *Cryptosporidium* remains a major health concern for animals and humans.
• Only Nitazoxanide received an agreement to treat human cryptosporidiosis.
• To tackle this parasite, it is mandatory to increase the therapeutic arsenal.
• Strategies to find new drugs ranged from drug libraries trials to drug repurposing.
• Targeting specific metabolic pathway in *Cryptosporidium* are promising approaches.

## Graphical abstract

**Figure:**
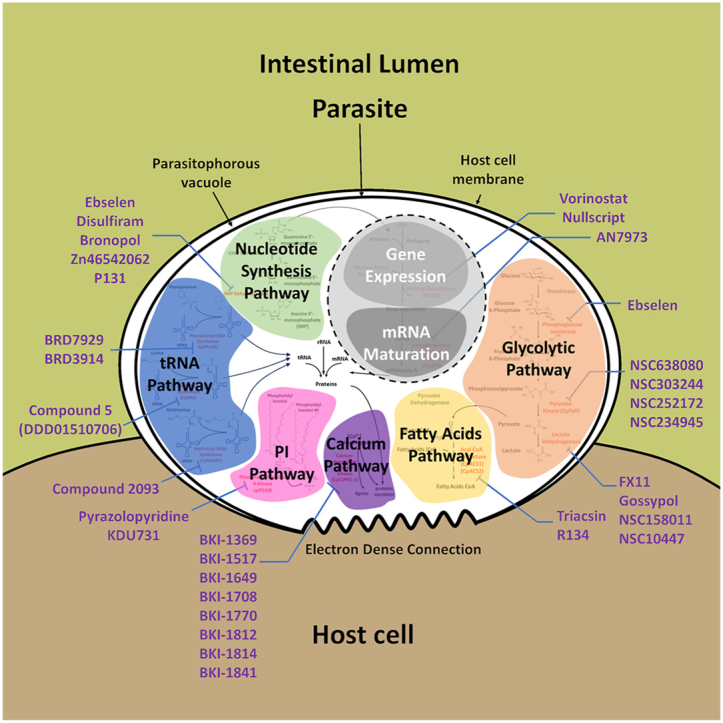

## 1 Introduction

*Cryptosporidium* is an intestinal parasite infecting a wide range of hosts including animals and humans. It is responsible for cryptosporidiosis, an infection that causes moderate to severe diarrhea, potentially fatal in young children and immunocompromised individuals (HIV/AIDS, transplant, chemotherapy). Forty-four species of *Cryptosporidium* have been documented so far (*Ryan* et al.*, 2021*) but *Cryptosporidium hominis* (*C. hominis*) and *Cryptosporidium parvum* (*C. parvum*) are responsible for more than 90% of human infections (*Feng* et al.*, 2018*). The global prevalence of *Cryptosporidium* infection in humans has been estimated at 7.6 % and the highest prevalence has been estimated at 69.6 % in some countries (*Dong* et al.*, 2020*). The most affected populations were from low-income countries, suffering from gastrointestinal disorders and located in rural areas. A meta-analysis study of long-term consequences associated with diarrhea from *Cryptosporidium* infection found *Cryptosporidium* was the fifth leading cause of diarrhea in children under 5, responsible for 48 000 deaths and a loss of 4.2 million disability-adjusted life years (*Khalil* et al.*, 2018*).

Although a vaccine has been developed for cattle (*Timmermans* et al.*, 2024*), no vaccine is available for humans. Regarding drug therapy, Nitazoxanide is the only drug approved for the treatment of cryptosporidiosis in immunocompetent patients. However, this medicine is not effective for immunocompromised adults and despite the use of a prolonged treatment, it exhibited low efficacy in treating HIV-infected children under 5 (*Amadi* et al.*, 2002*; *Amadi* et al.*, 2009*). There is therefore a critical need for new effective and affordable drugs. Drug discovery efforts for cryptosporidiosis have been hindered by the lack of cell cultures able to follow the long-term development of the parasite and the emergence of the complete sexual reproduction stages. The lack of robust screening assays and the complex life cycle have limited drug screening as well. Recent advances have been made to culture *Cryptosporidium* especially in three-dimensional (3D) environments like organoid systems to have a more physiologically relevant environment and to study the interaction between *C. parvum* and intestinal epithelial cells (*Bhalchandra* et al.*, 2020*). With the same objective, an intestinal epithelial stem cell-based platform enabling complete *Cryptosporidium* life cycle development was described using an “air-liquid” interface culture process (*Wilke* et al.*, 2019*). Interestingly, this method supported parasite expansion more than 100-fold and generated viable oocysts that were transmissible *in vitro* and *in vivo,* leading to infection and animal death in mice. Moreover, by the adaptation of hollow fiber technology mimicking the gut environment by the creation of an aerobic/anaerobic interface, it was possible to produce *Cryptosporidium* oocysts for more than 6 months. These oocysts were infective in a dexamethasone immunosuppressed mouse model (*Morada* et al.*, 2016*). However, not all these cell-based methods are compatible with High Throughput drug Screening (HTS) due to limitations including complexity, cost, or lack of automatization. So, 2D cell culture methods are still commonly used for HTS of drugs against cryptosporidiosis.

In this review, we will provide an overview of HTS scalable methods to quantify anticryptosporidial activity, the strategy based on metabolic pathway targeting, the repurposing/repositioning approach to speed up molecule availability and finally we will discuss challenges to take up in terms of molecule sources and future analytical tools.

## 2 How to quantify C. parvum development by HTS?

The traditional method for quantifying *Cryptosporidium* development is by microscopic examination involving counting the number of parasites or infectious foci per field of view by comparing the results to a reference molecule such as nitazoxanide or paromomycin. While this method is inexpensive, it is time-consuming and requires skilled personnel for accurate quantification (*Rochelle* et al.*, 2001*; *Teichmann* et al.*, 2012*). However, this approach is inconsistent with High Throughput Screening (HTS), a technique combining automated microscopy with image analysis to quantify multiple features of cellular events.

Indeed, recent advances in phenotypic assays adapted to HTS technologies have been made in direct parasite detection and are based on microscopic techniques using high content automatic imaging (Love et McNamara, 2020) or on biomolecular techniques.

One HTS method developed to assess inhibitory drug efficacy was based on indirect quantification of the *Cryptosporidium* parasite by cytopathic effect analysis. Chao and colleagues (*Chao* et al.*, 2018*) screened a collection of FDA-approved drugs leading to the confirmation of several previously known anti-*Cryptosporidium* hits as well as identifying a few new candidates. This model is simple, functional, and its homogeneous gain of signal format is amenable to High Throughput Screening. However, if one needs to confirm direct parasite activity, this procedure requires a secondary assay. The main advantage that phenotypic screening presents is that no special knowledge is required (for instance, the molecular target) to discover new molecules against *Cryptosporidium*. However, additional analyses are required to identify the targets or altered biological processes that might lead to side effects. In this context, target-based screening seems to be promising. The second HTS scalable strategy is based on a molecular biological approach. This explains the growing list of publications based on qRT-PCR methods (*Cai* et al.*, 2005*). More particularly, protocols have been adapted to HTS *in vitro* by the use of total cell lysate as a template (*Zhang and Zhu, 2015*). For example, based on these HTS methods, the drug screening of 1200 compounds allowed the identification of Vorinostat (SuberAniloHydroxamic Acid, SAHA) HDAC inhibitors against *C. parvum* (*Guo* et al.*, 2018*)*.* Additionally, 800 natural products were tested (*Jin* et al.*, 2019*) and led to the identification of active molecules such as cedrelone and baicalein. But more interestingly, these results provided a large selection of new structures derived from natural products, which could be explored as future therapeutics. In the same manner, *C. parvum* glucose-6-phosphate isomerase (*Cp*GPI) inhibitor: ebselen (*Eltahan* et al.*, 2018*) and several *C. parvum* hexokinase inhibitors (*Eltahan* et al.*, 2019*) were also identified by the use of qRT-PCR.

The third strategy of HTS scalable methods was based on genetically tractable C. parvum strains (*Vinayak* et al.*, 2015*). These transgenic strains have enabled researchers to develop more effective and faster drug screening methods by the use of *in vitro* luminescence assays (*Hulverson* et al.*, 2017*; *Hennessey* et al.*, 2018*). Recently, the use of such transgenic *C. parvum* strains (*Vinayak* et al.*, 2015*) in drug screening has been a real breakthrough, enabling the visualization of parasite development and the evaluation of drug efficacy with sensitivity and less variability. These strains can express fluorescent (GFP, RFP, mCherry) or bioluminescent reporters (luciferase). An *in vitro* bioluminescent test based on the use of the Nano-Glo luciferase reagent to quantify the luminescence emitted by the parasite has been used to study KDU731 *in vitro* and to monitor in real-time the parasite's infection by *in vivo* imaging using immunocompromised IFN-γ KO mice.

In parallel with the creation of new rapid screening methods, the diversification of leads and molecule sources needs to be increased. Indeed, the strategy to discover new anticryptosporidial drugs could be based first on the inhibition of molecules (mainly enzymes) previously identified as implicated in specific metabolic pathways. Another strategy to identify hits could be based on the screening of large drug libraries in a repurposing/repositioning strategy. Finally, a third strategy of drug sourcing could be achieved by the screening of natural compound libraries (for example plant- or marine-derived compounds). However, it is noteworthy that despite their efficacy against *Cryptosporidium*, many compounds exhibit toxicity. Considering this limitation, anticryptosporidial drug screening strategies will have to include additional chemical modification steps to produce safer analogs.

## 3 Targeting metabolic pathways: target-based screening

This approach consists first in identifying a metabolic pathway that is essential for *C. parvum* without interfering with the host. This strategy requires some knowledge of *Cryptosporidium*'s biological processes, such as metabolic pathways, which are extremely limited (*Abrahamsen* et al.*, 2004*).

A second prerequisite is to identify inhibitors of these metabolic pathways. This step is often completed by a repositioning/repurposing approach resulting in the assessment of the efficacy of inhibitors previously determined to be active against other pathogenic agents, but this time for *Cryptosporidium*. Such trials could be done directly on infected cell cultures or using recombinant target proteins. This last approach helped identify drug ability to bind and inhibit specifically target proteins.

Recent advances in the characterization of the genome and biology of *Cryptosporidium* have revealed that this parasite possesses distinct metabolic pathways and survival mechanisms that render it dependent on certain key proteins. *Cryptosporidium* is an obligate intracellular parasite with a minimalist metabolism and depends on the host for nutrient and energy supply (*Abrahamsen,* et al.*, 2004*; *Xu* et al.*, 2024*; Rider and Zhu, 2010).

Indeed, glycolytic and fatty acid pathways, nucleotide biosynthesis, mRNA maturation processes, protein synthesis, kinase activities were all investigated as potential target sites.

### 3.1 Targeting the energy metabolism of *C. parvum*

**Fig. 1:**
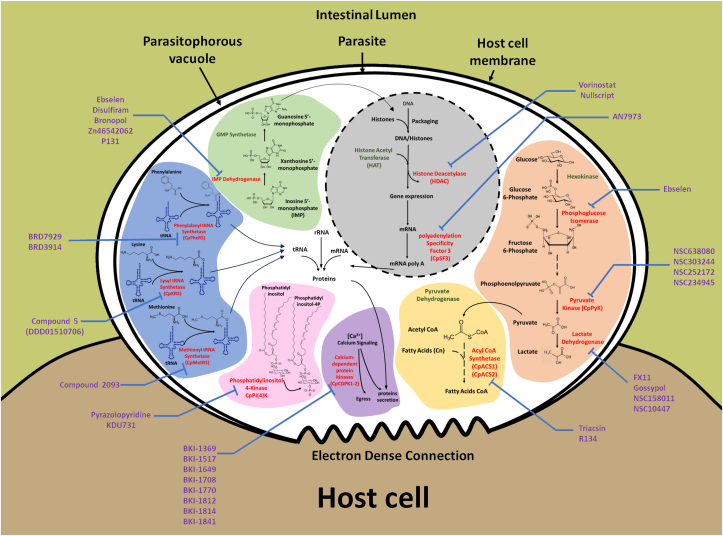
**Metabolic pathways targeted by anti-cryptosporidial drugs**. Several drugs against *Cryptosporidium* exhibit inhibiting effects on nuclear- (in grey) as well as cytoplasmic-located biochemical processes. In this figure, a *Cryptosporidium* parasite is represented in its intracellular but extracytoplasmic host location within the parasitophorous vacuole. Molecule exchanges between host cell and the parasite take place through the electron dense connection. Surrounding the parasite, the purple text refers to the name of compounds inhibiting specific metabolic pathways. In the nucleus, the histone acetylation process (*via* histone deacetylase) as well as mRNA maturation (*via* polyadenylation specificity factor 3) are both inhibited by drugs. In the cytoplasm the glycolytic pathway (in orange) could be impeded by the action on three different enzymes (phosphoglucose isomerase, pyruvate kinase and lactate dehydrogenase). Regarding the fatty acids pathway (in yellow), inhibitors were identified as acting on acyl-CoA synthetase). Calcium regulated processes (in purple) were also described as target sites by the inhibition of calcium dependent kinases. In the phosphatidyl inositol phosphorylating process, the inhibition of a kinase (CpPi(4)K) exhibited a strong activity by reducing parasite growth (in Pink). In the mechanism linking amino acids (phenylalanyl, lysyl or methionyl)) to their specific tRNA, actions of tRNA synthetases are also altered by therapeutic drugs (in blue). Finally, the guanosine synthesis pathway (in green) is inhibited by action on the inosine-5 monophosphate dehydrogenase. In red are the enzymes seen to be targeted by anticryptosporidial drugs. In order to make the figure readable, the glycolytic pathway has been reduced to the set of enzymes affected by anti-parasitic compounds. (For interpretation of the references to color in this figure legend, the reader is referred to the Web version of this article.)

Due to the absence of mitochondria (Riordan et al., 2003), *Cryptosporidium* cannot perform the Krebs cycle in its energy production, limiting its metabolic options for ATP production (Yu et al.*,* 2014; Rider and Zhu, 2010). Several studies (*Denton* et al.*,1996**;* Cook et al., 2015) have shown that *Cryptosporidium* predominantly relies on glycolysis, indicating that targeting this metabolic pathway could be considered as an interesting therapeutic option (Fig. 1). Similarly, lipid metabolism also plays a crucial role in the cellular functions of the parasite and *Cryptosporidium* is unable to synthesize certain essential fatty acids, such as long-chain fatty acids (*Zhu, 2004*). Therefore, several studies have focused on targeting pathways involved in the energy production of *C. parvum*.

#### 3.1.1 Glycolytic pathway

The glycolytic pathway has emerged as a promising target due to its essential role in ATP (Adenosine triphosphate) production and studies have shown that *C. parvum* relies mainly on the anaerobic oxidation of glucose for energy production. Several glycolytic enzymes such as phosphoglucose isomerase, pyruvate kinase (PyK) or lactate dehydrogenase (LDH) have been flagged as key enzymes for *C. parvum*'s development.

The Glucose-6-phosphate Isomerase described in *C. parvum* (CpGPI), catalyzing the interconversion of glucose-6-phosphate to fructose-6-phosphate, is also an interesting target in the glycolytic pathway. After the screening of 1200 known compounds to identify potential anti-CpGPI activities (Prestwick chemical library), the authors discovered that ebselen, a synthetic organoselenium compound, could irreversibly inhibit CpGPI with an IC_50_ of 8.33 μM without interfering with host GPI. Using a qRT-PCR-based drug testing assay, the authors showed that ebselen was able to inhibit the growth of *C. parvum in vitro* at micromolar level (EC_50_ = 165 μM). However, a relatively small safety interval was found, requiring further studies or investigating analogs to obtain more selective and efficacious inhibitors (*Eltahan* et al.*, 2018*).

Another enzyme acting in the *Cryptosporidium* glycolytic pathway was investigated as a potential drug target: the pyruvate kinase. This enzyme catalyzes the conversion of phosphoenolpyruvate to pyruvate. To test specific inhibitors, researchers have developed a recombinant *C. parvum* pyruvate kinase protein (CpPyK) and used it to screen 1424 compounds to identify potential CpPyK inhibitors (*Khan* et al.*, 2022*). Thanks to this strategy six compounds exhibited a concentration-dependent inhibitory effect (EC_50_ values ranging from 10.29 to 86.01 μM) on the *in vitro* development of *C. parvum*. Based on *in vitro* results (resulting in the assessment of efficacy and selectivity of tested drugs), these authors have selected 4 compounds: NSC638080, NSC303244, NSC252172 and NSC234945 for in vivo assays in IFN-γ KO mice. At a dose of 10 mg/kg body weight, NSC252172 and NSC234945 inhibited cryptosporidiosis in immunocompromised mice. The treatment induced very significant reduction in parasite load and in intestinal pathologies.

A third enzyme implied in the glycolytic pathway was finally studied: the lactate deshydrogenase (LDH). LDH catalyzes the interconversion of pyruvate to lactate and generates ATP. Previous studies have shown that *C. parvum*'s LDH (CpLDH) is essential for the parasite's growth by associating with the parasitophorous vacuole membrane for intracellular *C. parvum* development *(**Zhang* et al.*, 2015**)* and is essential for viability and infectivity of the shed oocysts in mice (*Zhang* et al.*, 2018*). Known inhibitors of LDH (gossypol and FX11) are effective against *C. parvum* but they are not specific to CpLDH and inhibit mammalian LDH as well. Recently, novel LDH inhibitors have been found: compounds NSC158011 and NSC10447 were identified for their anticryptosporidial properties using *in vitro* enzymatic assays by studying their inhibitory effects on recombinant CpLDH protein in HCT-8 cells. Analysis of cultures at 48h post-infection showed that NSC158011 and NSC10447 significantly inhibited the growth of *C. parvum* with IC_50_ of 14.88 μM and 72.65 μM respectively. In an *in vivo* model using IFN-γ KO mice, NSC158011 dosed at 400 mg/kg for 7 days showed comparable efficacy to paromomycin (100 mg/kg) on oocyst excretion, whereas a dose of 1000 mg/kg was required for NSC10447. Histopathological examination performed on the 9th day post-infection showed that mice treated with CpLDH inhibitors retained intact intestinal mucosa and villi, unlike untreated infected mice (*Li* et al.*, 2019*).

It is noticeable that recent results showed a strong synergistic effect against *Cryptosporidium* reproduction when two inhibitors of CpPyK and CpLDH are associated (*Khan* et al.*, 2023*) both *in vitro* and *in vivo*. Following the administration of NSC303244 + NSC158011 (100 mg/kg) or NSC252172 + NSC158011 (150 mg/kg), *C. parvum*-infected IFN-γ KO mice shed lower oocyst doses compared to mice treated with individual compounds. The double compound treatment completely cleared the infection without relapse.

#### 3.1.2 Fatty acid pathway

Unlike *Plasmodium*, *Toxoplasma*, and *Eimeria* apicomplexans, *Cryptosporidium* does not possess Type II fatty acid synthetic enzymes, suggesting this parasite is unable to synthesize fatty acids *de novo*.

However, *C. parvum* possesses a giant Type I fatty acid synthase (CpFAS1). This enzyme makes very long chain fatty acids using medium or long chain fatty acids as precursors (*Zhu* et al.*, 2000*). Indeed, this strategy of targeting lipid metabolism was used to discover triacsin C, an inhibitor of long-chain acyl-CoA synthetases (ACSLs), which are essential enzymes for fatty acid metabolism. The *C. parvum* genome encodes only three Acyl-CoA synthetases (CpACS) (*Zhu, 2004*). By colorimetry, *Guo and colleagues (**Guo* et al.*, 2014**)* characterized the enzymatic activity of the recombinant proteins CpACS1 and CpACS2 and demonstrated that inhibition of these proteins by triacsin C could decrease parasite growth, both in cell culture (IC_50_ = 136 nM) and in IL-12 KO mice where the excretion of oocysts was reduced by 50%–88% with doses of 8 mg/kg/d to 15 mg/kg/d for one week. More recently, triacsin C was used as a baseline to find analogs by virtual screening, resulting in the identification of molecule R134 as the best hit. However, these results are based on predictions that must first be tested in an *in vitro* model (Chattopadhyay et al., 2019).

### 3.2 Targeting the biosynthesis of nucleotides

The biosynthesis of nucleotides was initially identified as an attractive target, in particular a key enzyme involved in the *de novo* biosynthesis of guanine nucleotides: Inosine 5′-monophosphate dehydrogenase (IMPDH). The inhibition of IMPDH leads to impaired *Cryptosporidium* replication and survival. However, IMPDH finally proved to be an edifying example of the difficulty of working on *Cryptosporidium* metabolism pathways, faced with this parasite's incredible capacity for adaptation.

IMPDH is a key enzyme in the guanine nucleotide biosynthesis pathway in *Cryptosporidium.* Previous studies have suggested that inhibiting IMPDH in *C. parvum* (CpIMPDH) (*Umejiego* et al.*, 2004*) leads to a decrease in DNA and RNA production, blocking the replication and the survival of the parasite. Following i*n vitro* assays on infected HCT-8 cells, phylomers exhibited a parasite growth inhibition activity (phylomers 8, IC_50_ = 8 μM) (*Jefferies* et al.*, 2015*). Indeed, IMPDH inhibitors like P131 (250 mg/kg of body weight/day) exhibited in IL-12 KO mice a higher inhibitory effect against *Cryptosporidium* than nitazoxanide and paromomycin. (*Gorla* et al.*, 2014*).

Recently, a chemiluminescence-based HTS system was used to screen 1400 known bioactive compounds for any CpIMPDH inhibitory activity. Finally, three compounds - disulfiram, bronopol and ebselen - were identified. However, the selectivity of disulfiram and bronopol seemed not to distinguish enough between mammalian and protozoan IMPDH. Moreover, bronopol showed significant toxicity in mice (*Sarwono* et al.*, 2019*). These results encourage researchers to test analogs to overcome toxicity and specificity issues using virtual screening. Indeed, the compound ZINC46542062 was selected from a pharmacophore-based virtual screening to identify new inhibitors against CpIMPDH using P131 as a starting point, making it possible to consider a more specific activity and safety profile compared to the parent compound P131 (*Omolabi* et al.*, 2021*). In this context, other analogs have recently been discovered. After screening the Hokkaido University Chemical Library and using a luciferase-based High Throughput Screening method, researchers found two cyclophane-type adenosine derivatives having p-quinone amide moieties (compound 1 and 2) as weak inhibitors of CpIMPDH. To obtain more potent inhibitors, they synthesized four new derivatives free from cyclophane rings (compounds 3 to 6) (*Shigetomi* et al.*, 2019*). However, despite all these promising results, the use of directed gene ablation raised the question of using the nucleotide biosynthesis pathway as a good means of tackling the *Cryptosporidium* parasite (*Pawlowic* et al.*, 2019*). It was particularly interesting to discover that resulting parasite mutants were viable under normal conditions but were hypersensitive to inhibition of purine nucleotide synthesis in their host cell. This observation suggested that the lack of purine synthesis capacity in the parasite was compensated for by salvage enzymes or yet undiscovered purine transporters. Therefore, these results clearly highlight the importance of considering the parasite's capacity to bypass metabolic pathway inhibition by molecule uptake directly from the host cell.

### 3.3 Targeting nucleic acid processes and gene expression

Within the nucleic acid metabolism pathway, two modification steps were targeted for drug screening. The first one aimed at the epigenetic regulation process by the acetylation of histones. The second target focused on the addition of the polyadenylation tail to the mRNA during the post transcriptional maturation step.

Thus, Vorinostat, a molecule previously approved by the FDA for the treatment of cutaneous T-cell lymphomas was investigated for its inhibitory effect on histone deacetylases (HDACs) in *Cryptosporidium*. Interestingly, HDACs are a family of enzymes that remove acetyl groups from histones and play a role in gene expression by modifying the level of DNA compaction. Using the qRT-PCR-based phenotypic screening assay Vorinostat was shown to be effective in inhibiting *C. parvum* histone deacetylase 3 (CpHDAC3). *In vitro* studies have shown inhibition of parasite growth, with an EC_50_ of 0.203 μM. In *in vivo* models of immunocompromised mice (IL-12 KO mice) infected with *C. parvum*, a dose of 25 mg/kg/day reduced oocyst excretion after 6 days of treatment (*Guo* et al.*, 2018*; *Herrera-Martínez* et al.*, 2020*). In this context, and just like Vorinostat, another HDAC inhibitor molecule has recently been discovered in an epigenetic library of compounds already approved by the FDA: nullscript. The authors have demonstrated that nullscript inhibits the growth of *C. parvum* by inhibiting the deacetylation of histone 3 (H3K9). Its effectiveness has been demonstrated *in vitro* with a IC_50_ of 2.1 μM on infected HCT-8 cells and *in vivo*, in SCID mice whose oocyst excretion significantly decreased after three days of treatment at 10 mg/kg/day (*Murakoshi* et al.*, 2020*).

In parallel with the HDAC inhibition strategy, the mRNA maturation process was also investigated as a potential drug target. In this approach, AN7973 was identified through a drug repositioning strategy by screening a library of 7802 compounds, including analogs to four antiprotozoal chemical scaffolds under pre-clinical development for neglected tropical diseases. *In vitro*, in the *Cryptosporidium*-MDCK cell growth model, AN7973 selectively inhibited the intracellular development and more specifically the asexual reproduction of *C. parvum* (IOWA) and C. *hominis* (TU502) by inhibiting DNA synthesis, probably by targeting CPSF3 (Cleavage and Polyadenylation Specific Factor 3). In an *in vivo* model, a 4-day treatment on the 7th day post-infection reduced oocyst excretion by more than 90% at 10 mg/kg/day and over 99% at 25 mg/kg/day in immunocompromised mice (NOD SCID Gamma). A dose dependent efficacy was also demonstrated for AN7973 in IFN-γ KO mice (efficacy at a dose of 10 mg/kg/day. In addition, its effectiveness has also been demonstrated in newborn calves without side effects, encouraging the continuation of preclinical toxicological studies (*Lunde* et al.*, 2019*).

### 3.4 Targeting protein synthesis

Before translation steps leading to the production of proteins, tRNA are associated with their specific amino acids using tRNA synthetase. Three of them were targeted during anticryptosporidial drug screening studies.

First of all, several bicyclic azetidine compounds with known mechanisms of action against the phenylalanyl-tRNA synthetase on *Plasmodium falciparum (pf*PheRS*)* were tested *in vitro* against the *Cryptosporidium* enzyme exhibiting the same function *(*CpPheRS*).* Following a cell-based high-content microscopy assay *(**Vinayak* et al.*, 2020**)* the bicyclic azetidine BRD3914 showed the most potent *Cryptosporidium* inhibitor activity with an EC_50_ of 62 nM. Further characterizations of a selection of bicyclic azetidines and their synthetized analogs have demonstrated their efficacy against different strains and species of *Cryptosporidium* and their safety. Among the multiple hits, the bicyclic azetidine BRD7929 showed significant efficacy curing immunosuppressed mice (NOD SCID Gamma) at doses at low as 10 mg/kg/day for 4 days without relapse after 14 days’ observation. To directly test if the mechanism of action was conserved, the authors used CRISPR/Cas9 genome editing to introduce mutation in CpPheRS (L482V) and showed that the mutant transgenic parasite showed a 23-fold decrease in BRD7929 susceptibility and 9-fold in BRD3914 susceptibility. The molecular target (PheRS) was confirmed using recombinant *C. hominis* PheRS (ChPheRS) protein, showing that inhibition of the aminoacylation activity of recombinant ChPheRS is correlated with *in vitro* activity in culture and validating *Cryptosporidium* PheRS as the molecular target of bicyclic azetidines (*Vinayak* et al.*, 2020*).

A previous study showed BRD7929 blocked nuclear replication using EdU (5- ethynyl- 2′- deoxyuridine) incorporation and specific monoclonal antibodies. The authors demonstrated that parasites in control cultures had progressed to the mature meront stage but only trophozoites were present in BRD7929-treated cultures (*Funkhouser-Jones* et al.*, 2020*).

The *C. parvum* methionyl-tRNA synthetase (CpMetRS) was also investigated as a potential drug target and resulted in identifying the compound 2093 as an inhibitor. This compound showed significant inhibitor activity against different strains of *C. parvum* in HCT-8 cells with an EC_50_ ranging from 6 nM to 29 nM. *In vivo* assays using IFN-γ KO mice (*Buckner* et al.*, 2019*) confirmed this parasite growth inhibitory effect following a treatment with 50 mg/kg twice a day. However, the compounds revealed a resistance issue (*Hasan* et al.*, 2021*). The authors evaluated the efficacy of these compounds in calves infected by *C. parvum*. The treatment of 15 mg/kg every 12 h, starting on the second day of infection, made it possible to considerably reduce the excretion of oocysts. Unfortunately, a gradual increase in excreted oocysts was observed as well as a reappearance of diarrheal symptoms in two of the three calves treated. After sequencing, the authors noticed that the two relapsed calves were infected with parasites presenting mutations in the gene coding for CpMetRS. Two mutations involved in changing amino acids (D243E and T246I) conferred resistance to the drug. Structural modeling indicated that these mutations strongly disrupt inhibitor binding to CpMetRS. Subsequently, tests carried out with recombinant enzymes containing these mutations showed that they were 170 times less sensitive to the inhibition of Compound 2093. Similarly, *C. parvum* parasites genetically modified by the CRISPR/Cas9 method to express these mutations were 128 times (D243E) and 613 times (T246I) less sensitive to the 2093 inhibitor (*Hasan* et al.*, 2021*).

This study reinforces the urgent need to develop new therapeutic molecules and the concern about the rapid resistance of *Cryptosporidium* to drugs.

Finally, Lysyl tRNA synthetase (KRS) was also identified as a potential drug target against *Cryptosporidium*. Initially this enzyme was identified in *Plasmodium falciparum* as a target of a fungal secondary metabolite: cladosporin (*Hoepfner* et al.*, 2012*). Unfortunately, cladosporin exhibited a high metabolic instability leading to this compound being considered a poor drug lead. Despite this limitation, based on 96% structural homology, *pf*KRS inhibitors were still tested on *Cryptosporidium* Lysyl tRNA synthetase (CpKRS) activity (Baragaña et al.*, 2019*). Using a kinase glo assay platform, a 13 000-compound library (Tres Cantos Antimalaria Set, GlaxoSmithKline) was screened for compounds to use against recombinant KRS. A chromene compound (N-(cyclohexylmethyl) 4-oxo-4H-chromene-2-carboxamide called Compound 2) was identified as an inhibitor and modified to increase its stability as well as oral bioavailability. This optimized lead (N-[(4,4-difluoro-1-hydroxy-cyclohexyl) methyl]-6-fluoro-4-oxo-chromene-2-carboxamide (called compound 5 or DDD01510706) inhibited *in vitro* development of both *C. hominis* (TU502) with an EC_50_ = 6 μM and *C. parvum* (IOWA strain) with an EC_50_ = 1.3 μM). Compound 5 exhibited excellent drug-like properties by competing with the ATP for the same bonding site on CpKRS. Interestingly, Compound 5 exhibited *in vivo* activity against *Cryptosporidium* in NOD SCID and in INF-γ KO mice treated orally at 20 mg/kg once a day for 7 days. Following the validation of CpKRS as the molecular target of the chromene-based compound, the mode of action was confirmed (*Hanna* et al.*, 2023*). Using a conditional CRISPR/Cas9 knockdown strategy, CpKRS was proved to be essential for *Cryptosporidium* infection in IFN-γ KO mice. Interestingly, the overexpression of CpKRS or the mutation of CpKRS's active site supported resistance to treatment. Indeed, it provided important validation of KRS as a drug target.

### 3.5 Targeting protein kinases

As shown above, targeting single pathways or a single component within one pathway is not a robust enough strategy due to the ability of the parasite to find alternative pathways or develop resistance. Targeting multiple metabolic pathways is therefore a much better approach.

Calcium-Dependent Protein Kinases (CDPKs) have emerged as promising candidates due to their essential role in parasite development and lack of counterparts in mammalian hosts (*Ojo* et al.*, 2010*). These kinases are involved in calcium signaling and regulate various processes such as invasion, motility, and egress. Recent studies showed that CpCDPK1 and CpCDPK9 might play different roles in the invasion and growth of *C. parvum*. They are transcribed at different times during development and are expressed in different organelles of the parasite. Therefore, CpCDPK9 is more likely to play a role in the invasion of *C. parvum*, while CpCDPK1 might participate in the growth of the parasite. Nevertheless, inhibition of both CDPKs in *in vitro* cultures reduced the parasite load (∼40%) (*Su* et al.*, 2022*). Also, gene encoding CpCDPK2A is one of the CDPK genes highly expressed in multiple life cycle stages of *C. parvum* and it is one of the first CpCDPKs that has been identified with high expression in macrogamonts, suggesting that it is a good target for the development of drugs against *Cryptosporidium spp* (*Shu* et al.*, 2022*).

In 2016, the screening of Bumped Kinase Inhibitors (BKIs) against *Cryptosporidium* both *in vitro* and *in vivo* models identified the compound BKI-1517 as an interesting drug candidate (*Castellanos-Gonzalez* et al.*, 2016*). The results showed that a concentration of 10 μM resulted in a 90% reduction in the number of parasites in HCT-8 cells infected with an EC_50_ ranging from 10 nM to 50 nM. In immunocompromised SCID mice, the authors evaluated the efficacy of BKI-1517 dosed at 60 mg/kg for 5 days, administered in a single or divided daily dose starting from the 4th day post-infection. Surprisingly, the 60 mg/kg/day administered as a single dose was more effective than the divided dose, but an increase in oocyst excretion was observed in several mice on the 28th day after the start of treatment. However, this relapse was limited by doubling the dose of BKI-1517, and 83.3% of the mice were cured.

A second inhibitor, BKI-1369, also showed good *in vitro* results with an EC_50_ of 2.4 μM and demonstrated efficacy in several animal models infected with *C. parvum*, for example calves infected and treated with 5 mg/kg/day of BKI-1369 at the 2nd day of infection. Results showed an oocyst excretion reduced up to 30-fold, and a decrease in diarrheal symptoms was observed as early as the 3rd day of infection (*Hulverson* et al.*, 2017*). In the gnotobiotic piglet model infected with *C. hominis* and treated with 10 mg/kg of BKI-1369 twice a day for 5 days (*Lee* et al.*, 2018*), the piglets showed a reduction in oocyst excretion throughout the study from the 2nd day of treatment. Similarly, unlike in the control group, diarrheal symptoms significantly decreased and even completely disappeared for some of the piglets treated. Histopathological analysis conducted on the 13th day post-infection in the control group showed structural abnormalities of intestinal villi, lymphocyte infiltration of the lamina propria, and a significant infection of *C. hominis* at the apical pole of epithelial cells. In contrast, in the group treated with BKI-1369, cellular damage, inflammation, and parasite quantity were greatly reduced.

However, the main drawback of BKI-1369 is that it blocks the hERG (Human Ether-a-go-go-Related Gene) ion channel, leading to cardiotoxicity at concentrations as low as 2 μM, thus limiting its use in clinical studies in humans.

Additional research has been carried out to find safe and effective drug candidates for *Cryptosporidium* by structure modification of BKIs using a pyrrolopyrimidine (PrP) central scaffold. Newly designed BKIs (BKI-1770 and BKI-1708) were found to exhibit efficacy against *C. parvum* without cardiotoxicity issues using *in vitro* and IFN-γ KO mouse models (*Huang* et al.*, 2019*). Similarly, new BKI-1812 and BKI-1814 were tested (*Hulverson* et al.*, 2021*) but the authors demonstrated that several human kinases were affected by both BKIs and BKI-1814 was quickly metabolized into high plasma concentration of its metabolite BKI-1649, a compound which showed abortifacient toxic effects in pregnant mice, limiting the development of these BKIs. Overall, the authors showed that data from these studies serve as a strong proof-of-concept for the continued development of the pyrrolopyrimidine (PrP) central scaffold as inhibitors of CpCDPK1 for the treatment of *Cryptosporidium* (*Hulverson* et al.*, 2021*). Other BKI analogs based on a 5- aminopyrazole- 4- carboxamide (AC) scaffold like BKI-1770 and BKI-1708 were tested and shown to be efficacious in the *C. parvum* infected IFN-γ KO mouse model, emerging as potential pre-clinical leads for cryptosporidiosis treatment (*Huang* et al.*,2019*). Finally, the authors highlighted the utility of knowing the metabolism of these BKI analogs, in particular the potential effects of their metabolites during a study comparing toxicities among BKI analogs (*Hulverson* et al.*, 2023*). Indeed, BKI-1770, BKI-1841 and BKI**-**1708 showed significant efficacy in *in vitro* and in mice models with different dose intervals to reach efficacy against *C. parvum* and these variations could be explained by different metabolic stabilities. Despite efficacy against *C. parvum*, BKI-1770 and BKI-1841 resulted in neurological and bone toxicity effects in calves and rodents. However, BKI-1708 continues to be a potential candidate for cryptosporidiosis treatment (*Hulverson* et al.*, 2023*).

Finally, another type of protein kinase was investigated as a potential anticryptosporidial drug target: Phosphatidyl inositol-4-kinase (CpPI(4)K). Following the screening of 6220 compounds against *C. parvum* with known activity against various protozoan parasites (*Plasmodium falciparum* and *Trypanosoma brucei*) using the high-content imaging infection assay previously described and a second screening based on cell viability (*CPE, Cytopathic Effect*), the authors found pyrazolopyridine analogs and other known PI(4)K inhibitors to be effective against *C. parvum* and against *C. hominis*. Notably, KDU731 exhibited inhibitor activity on CpPI(4)K by interacting with the ATP-binding site of the enzyme (*Manjunatha* et al.*, 2017*). *In vitro* assays using KDU731 confirmed the efficacy against *C. parvum* (EC_50_ = 0.1 μM) and *C. hominis* (EC_50_ = 0.13 μM). Oral treatment with Pyrazolpyridine KDU731 significantly reduced intestinal infection and oocyst shedding in a IFN-γ KO mice model (treated 7 days post infection with a dose of 10 mg/kg for 1 week) and neonatal calves (treated 4 days post infection with 5 mg/kg every 12h for 7 days).

A daily dose of 10 mg/kg of KDU731 for 7 days reduced the excretion of oocysts from infected mice. In addition, histological analyzes carried out at the end of treatment show the absence of parasites in the intestines of treated mice, whereas this is not the case in untreated animals. The authors then evaluated the efficacy of KDU731 in newborn calves at 5 mg/kg, showing a significant reduction in the excretion of oocysts, diarrhea, and dehydration, from three days of treatment (*Manjunatha* et al.*, 2017*).

## 4 Repositioning and repurposing strategy

During drug development, the biopharma industry or research laboratories often reposition agents for a different indication than what they were originally intended for. In other words, repositioning takes place during drug development before approval (for instance before FDA approval). By contrast, drug repurposing refers to the change in therapeutic indication for an established drug (*Ballard* et al.*, 2020*). Considering these definitions, several drugs active against a broad spectrum of pathogenic agents were tested for their anticryptosporidial activity (Bessoff et al., 2013, 2014; *Guo* et al.*, 2018*). As indicated in the previous part, the repositioning/repurposing strategy was often used when a specific metabolic pathway was targeted and led for instance to the identification of CpPI(4)K inhibitors, CpKRS inhibitors or HDAC inhibitors (like Vorinostat). Nevertheless, repurposing/repositioning is not always successful. Indeed, despite trials with medicines used to treat HIV positive patients (such as spiramycin (*Wittenberg* et al.*, 1989*), clofazimine (Iroh Tam et al., 2021), azithromycin (*Blanshard* et al.*, 1997*), clarithromycin (*Fichtenbaum* et al.*, 2000*), or paromomycin (*Fichtenbaum* et al.*, 1993*)), clinical success has not yet been achieved.

However, a repositioning/repurposing approach remains of major interest to researchers because it drastically reduces efforts to synthesize drugs and in fact it represents a relevant way to accelerate the discovery of new treatments in a cost-effective manner.

Moreover, these compounds can easily be tested to obtain pharmacokinetic and ADMET (Absorption, Distribution, Metabolism, Excretion, and Toxicity) parameters.

For example, in 2017, a large library of 78 942 compounds was screened and clofazimine, an FDA-approved drug for treating leprosy, was identified with sub-micromolar activity against the two most common *Cryptosporidium* species infecting humans: *C. parvum* and *C. hominis* (*Love* et al.*, 2017*). However, in a Phase 2A clinical trial, the efficacy of clofazimine for the treatment of cryptosporidiosis failed in a severely immunocompromised HIV patient (Iroh Tam et al., 2021).

Using the same strategy, two hit compounds were found after the screening of an open-access drug repurposing library named ReFRAME (*Repurposing, Focused Rescue, and Accelerated MedChem*) comprising over 12 000 compounds of which 38% were FDA-approved drugs and 59% previously tested in clinical trials. VB-201 and ASP-7962 showed an *in vitro* efficacy against *C. parvum* (EC_50_ 860 nM and 54 nM, respectively). *In vivo,* a significant reduction of oocyst shedding (100-fold and 10-fold for VB-201 and ASP-7962, respectively) was observed in juvenile IFN-γ KO mice when drugs were administrated at 10 mg/kg and 50 mg/kg twice per day for 3 day at 4 days post-infection. It is noteworthy that these results were better than the standard treatment with nitazoxanide (*Janes* et al.*, 2018*). However, the mechanisms by which these compounds exhibit anti-cryptosporidial activity is not yet known and require further investigation.

The repurposing/repositioning strategy led also to the identification of a piperazine-based inhibitor, compound MMV665917, as a good candidate to treat cryptosporidiosis. In an *in vitro* HCT-8 model, EC_50_ values ranging from 2.10 μM to 4.05 μM were determined for different species (*C. parvum* and *C. hominis* strain TU502) (*Jumani* et al.*, 2018*). Using specific fluorescent antibodies for each stage of the parasite, it was demonstrated that compound MMV665917 particularly inhibited the development of macrogamonts (*Funkhouser-Jones* et al.*, 2020*). In NGS mice, the efficacy of compound MMV665917 was evaluated on the 7th day post-infection at 30 mg/kg/day or 60 mg/kg/day and compared to the efficacy of paromomycin (administered at 1000 mg/kg/day) for 7 days of treatment. As with treatment with paromomycin, oocyst excretion was reduced by over 90% in the presence of compound MMV665917, but a relapse was observed at the dose of 30 mg/kg (although not at the dose of 60 mg/kg) (*Jumani* et al.*, 2018*). The efficacy of MMV665917 was also determined in newborn calves infected with *C. parvum* oocysts. A daily dose of 22 mg/kg was administered two days after the onset of severe diarrhea and continued for 7 days. The treatment significantly reduced the severity of diarrhea and the quantity of excreted oocysts (94%) (*Stebbins* et al.*, 2018*). Additionally, promising results were obtained in gnotobiotic piglets (*Lee* et al.*, 2019*). However, a low inhibition of the hERG ion channel, correlated with cardiotoxicity, was reported. Recently, a new inhibitor has been developed through optimization of MMV665917 to reduce toxicity, the triazolopyridazine compound SLU-2633 which has an EC_50_ of 0.17 μM and is 12 times more potent than MMV665917. It demonstrated significant efficacy in NSG mice in just 4 days at 50 mg/kg BID (*Oboh* et al.*, 2021*). Analogs of SLU-2633 have been synthetized and tested to reduce their affinity to the hERG channel but for the moment all the compounds have been less potent and future studies are necessary (*Oboh* et al.*, 2023*).

## 5 Discussion

Despite the numerous challenges associated with the development of drugs against *Cryptosporidium*, significant progress has been made in identifying new therapeutic molecules that demonstrate both *in vitro* and *in vivo* activity. Several dozen compounds have been tested in various animal models, with the most advanced ones including BKI-1369, compound 2093, KDU731, and MMV665917.

Historically, traditional screening methods were primarily based on manual analyses using microscopy or molecular methods to quantify the development of *C. parvum* in the presence of inhibitors. The advent of High Throughput Screening techniques has enabled the automation of many screening steps, rapidly analyzing many compounds while obtaining quantitative and more reproducible data. Two types of strategy have been employed to screen for anti-*Cryptosporidium* molecules: the phenotypic approach has directly tested the effect of compounds on the parasite itself by observing phenotypic changes. This method has been used particularly when the biological target has not yet been clearly identified and allows new targets to be discovered. However, additional analyses are required to identify the targets or altered biological processes that might lead to side effects. Contrary to the phenotypic approach, the target-based approach involves identifying and then validating specific targets of the parasite. The goal is to use compounds that will selectively interfere with the parasite's metabolic pathways (energy metabolism, replication, and other pathways essential for the parasite's survival) while minimizing their effects on the host cell.

### 5.1 Why is targeting a metabolic pathway of *C. parvum* so challenging?

Several studies highlight the difficulty in targeting certain metabolic pathways or enzymes because the metabolic pathways of parasites are often similar to those of host cells, making it difficult to create specific drugs for the parasites without harming the host. Targeting the parasite's metabolic pathways is therefore particularly complex. As a matter of fact, the metabolic pathways of *C. parvum* are often less well-characterized compared to those of the hosts, which makes the selection of specific metabolic targets more challenging, and many inhibitors have multiple modes of action. For example, ebselen, an inhibitor initially discovered as an anti-GPI, showed a new mechanism of action after a repurposing drug strategy, and demonstrated an inhibition action against CpIMPDH. In this study, ebselen seemed to be of a mixed-model inhibitory mechanism that changes from a reversible mode to an irreversible mode depending on the concentration of ebselen (*Sarwono* et al.*, 2019*).

Moreover, recent studies clearly demonstrated the parasite's ability to circumvent the sudden absence or inactivation of such a pathway. Research on IMPDH was particularly edifying, as Pawlowic's findings (*Pawlowic* et al.*, 2019*) proved that this enzyme was not essential for *Cryptosporidium*. The authors suggested that mutant parasites lacking this pathway remained viable due to their ability to transport purine *via* an unknown transporter or to access an as yet undiscovered rescue enzyme. Following these observations, and despite much research targeting IMPDH as a promising target, IMPDH should be considered a poor drug target. In the same vein, the inactivation of *Cryptosporidium* hexokinase (an enzyme that catalyzes the first reaction of glycolysis) had no impact on the parasite growth. These results suggested that *Cryptosporidium* utilizes an alternative pathway to obtain phosphorylated glucose and also highlighted the metabolic flexibility of *Cryptosporidium*. (*Xu* et al.*, 2024*). Furthermore, studies on compound 2093, an inhibitor of CpMetRS, revealed a resistance issue and exemplify the fragility of strategies based on specific targets.

Such observations of *Cryptosporidium* 's ability to develop spontaneous resistance highlighted the importance of improving High Throughput Screening techniques and refining *in vitro* tests to quickly identify resistance phenomena. In parallel with this methodological innovation, an alternative way to circumvent the issue raised by parasite adaptation/resistance would be to treat patients with several inhibitors. As demonstrated by Khan and colleagues (*Khan* et al.*, 2023*), in some cases a synergistic efficacy can be observed, but this double treatment will also reduce the capacity for the parasite to generate mutations leading to resistance.

Another way to limit the emergence of resistant parasites could be to develop therapeutic strategies based on the enhancement of host cell defense. For instance, the screening of 473 kinase inhibitors found three compounds exhibiting selective targeting of tyrosine kinases. The authors point out that *Cryptosporidium* does not express any tyrosine kinases, but its genome encodes three tyrosine kinase-like enzymes (TKLs). Future studies will be needed to determine the role of these TLKs using genetic approaches to confirm the target. Also, the authors discovered that several kinase inhibitors enhanced parasite growth, suggesting that host kinases may be involved in preventing *Cryptosporidium* infection (*Nava* et al.*, 2023*). In this context, a recent study examined the role of protein kinase C-α (PKCα) activity in human HCT-8 intestinal epithelial cells during infection with *C. parvum* sporozoites. Their results show that HCT-8 cell PKCα is activated by *C. parvum* infection and plays an important role in promoting parasite adherence and invasion. Their findings suggest intestinal epithelial cell PKCα as a potential host-directed therapeutic target for cryptosporidiosis. By specifically targeting host mechanisms, host-directed therapies can have potential advantages, such as reducing the risks of parasite resistance emergence (*McCowin* et al.*, 2022*).

Another limitation observed during drug development targeting on a specific metabolic pathway is to consider if the targeted set of enzymes is active during all parasite life stages. Thus, a stage-specific gene expression has been shown in the energy metabolism of *C. parvum*, (in particular the glycolytic pathway, where the enzymes are not expressed in the same way during the *C. parvum* life cycle (*Tandel* et al.*, 2019*)). This calls into question the targeting of the glycolytic pathway as a potential drug target. Additionally, as for glycolytic enzymes, CpACS genes involved in fatty acid metabolism were differentially expressed at different parasite developmental stages, also with different subcellular localization. Indeed, CpACS1 is mainly responsible for the fatty acyl-CoA synthesis during the parasite invasion and/or early stage of intracellular development whereas CpACS2 is active throughout the entire parasite life cycle (*Guo* et al.*, 2016*). Therefore, targeting a specific enzyme that is present throughout the life cycle remains complex.

From a general point of view, drug discovery is also limited by the appearance of side effects in animal models that could represent major obstacles for medicine approval. In this context, the example of CDPK1 inhibitors (such as BKI-1534 and BKI-1649) mentioned previously was particularly revealing. Indeed, both molecules failed the mouse pregnancy safety test despite their excellent efficacy in treating *Cryptosporidium* infection in mice. As this assay was mandatory for pediatric safety approvement by the FDA, this failure eliminated both molecules from further consideration. The study of BKI-1369 has further highlighted this side effect issue and discussed the importance of final molecule concentration detected in the plasma. So, daily administration in mice of BKI-1369 at a dose of 300 mg/kg led to a plasma concentration of 8.3 μM, and signs of cardiotoxicity were demonstrated in Q-Patch, Q-TiSA and rat cardiovascular assays. However, administration of the same compound at a dose of 150 mg/kg led to a final concentration in plasma of 3.9 μM and no sign of toxicity was observed in animal models. These results led to BKI-1369 being considered as a viable candidate for animal use when administered at a low concentration. During high systemic exposure however, the potential cardiotoxicity of BKI-1369 could hinder its development as a potential treatment for humans.

This example of BKI studies was nevertheless particularly interesting as it raised the question of the final treatment strategy in terms of systemic versus nonsystemic use. This question of systemic action for future therapeutics against *Cryptosporidium* is crucial. The review by Zhu and colleagues (*Zhu* et al.*, 2021*) highlighted several complex questions. First of all, the absorption of a drug could be reduced in a diarrheal context that may flush out the therapeutic compound. In order to limit this, future treatment based on systemic drugs could be administrated at the same time as an anti-diarrheal medicine. Secondly, following the absorption step, systemic drugs will need to be able to pass through the electron dense band. Because of its highly “selective” properties, the ED band could impede drug access to the parasite imbedded in the parasitophorous vacuole. Consequently, future screening processes will require assessment of the ED permeability for hits/leads. In some cases, nonsystemic drugs could represent an alternative therapeutic strategy. This approach could be of interest for compounds featuring poor absorbability. If *Cryptosporidium* had been present uniquely in the gut, a systemic exposure would not have been required as was observed for BKI efficacy (*Arnold* et al.*, 2017*). In this case, the action of a drug located only in the gut would be expected to minimize toxicity associated with systemic drug distribution. One should consider the possibility of screening drugs active in the digestive tract and which may be eliminated by passing through the liver. These ‘soft drugs’ would also be of interest in the reduction of toxic side effects and be useful to treat *Cryptosporidium* in immunocompetent adults or children. But this does not take into consideration the fact that the small intestine is a major site (besides the liver) for drug biotransformation by Cytochrome P450 metabolism (Thelen et al., 2009). However, *Cryptosporidium* is not always located in the gastrointestinal tract. In people with AIDS, *Cryptosporidium* was observed in the respiratory tract indicating the necessity of a systemic action drug (*Meynard* et al.*, 1996*). This question of the systemic action of future drugs was also raised by Arnold *(**Arnold* et al.*, 2017*). The authors highlighted the need for future drugs to treat *Cryptosporidium* not only through good exposure in the gastrointestinal tract but also through systemic exposure. Thus, future screening strategies will have to integrate the metabolization processes of the therapeutic compounds and their diffusion in plasma.

### 5.2 Natural products, a new source of anti-*Cryptosporidium* compounds?

Considering issues limiting drug screening strategies, finding innovative compounds is also a major obstacle in anticryptosporidial drug discovery. As previously described in the repurposing/repositioning strategies, several synthetic molecular banks have already been tested, but active molecules purified from plant extracts have not yet been fully explored. Indeed, the main advantage of natural products is that they present a diverse range of bioactivities and provide a set of new chemical structures which could prove to be a source of new, so far unidentified anticryptosporidial hits (for review see *Ali* et al.*, 2024*). Furthermore, these products might be more easily allowed in animal feed (for example) compared to synthetic drugs. A library of 3764 marine-derived compounds was screened to discover new inhibitors against *C. parvum*. After selecting 23 compounds and purifying them, the macrolide leiodolide A, derived from a sponge of the genus *Leiodermatium*, was tested, and showed very good selectivity for the parasite in HCT-8 cells, as well as an EC_50_ of 103.5 nM (*Bone Relat* et al.*, 2022*). This is the second marine-derived compound identified for its anticryptosporidial activity after tartrolon E, a compound from shipworm symbiotic bacteria (*Teredinibacter turnerae*). This has been shown to display effective activity against a broad range of apicomplexan parasites including *C. parvum, in vitro* (EC_50_ = 3.85 nM) and *in vivo* in a neonatal mouse model with no signs of toxicity up to 40 mg/kg (*O'Connor* et al.*, 2020*). Tartrolon E has a rapid inhibitory action on asexual stage parasites and prevents establishment of infection, with an additional inhibitory action on early sexual development during the transition to sexual development (*Cotto-Rosario* et al.*, 2022*). Compounds derived from plants have been discovered in *The NatProd Collection* (800 pure chemicals derived from natural products) using a qRT-PCR High Throughput Screening. This screening identified three top phytochemicals: cedrelone (EC_50_ = 0.267 μM; SI = 13.4), the homoisoflavanoid Deox B 7,4 (EC_50_ = 0.734 μM) and baicalein (EC_50_ = 0.981 μM), which produced long-term inhibition on the intracellular parasites (*i.e*, irreversible killing) despite a relatively short treatment time. Among them, cedrelone and baicalein inhibited the parasite growth in a time-dependent manner, whereas Deox B 7,4 produced a “delayed death effect” on *C. parvum* (*Jin* et al.*, 2019*). These results support the need to determine the effective treatment window for the best results against *Cryptosporidium*.

### 5.3 Innovative tools for drug screening against *Cryptosporidium*

With regard to the tools currently available to assess drug efficacy using rapid, sensitive and easy-to-use methods, several limitations have been observed and innovative approaches have been developed to circumvent these problems. Firstly, the cell culture models used to develop the parasite still need to be improved. Secondly, new analytical processes have to be developed and adapted to enable High Throughput Screening.

Initially a major issue in *in vitro* drug screening assays came from the lack of highly efficient cell culture methods supporting both sexual and asexual parasite stages. Several recent major improvements in cell culture (by the use of hollow fiber (*Morada* et al.*, 2016**)*, 3D silk scaffolds (*Cardenas* et al.*, 2020*), colo680N cells (*Miller* et al.*, 2018*), primary cell, stem-cell-derived IEC under ALI conditions (Wilke et al., 2019, 2020) or organoids (*Heo* et al.*, 2018*)) have suppressed these technical limitations thanks to their ability to sustain the complete parasite development (including the fertilization step). But one should consider these technical approaches as a black box: not user-friendly and unsuited to investigating drug activity at the cell level and consequently, not scalable to HTS.

Moreover, when cell culture supports all life stages, *C. parvum in vitro* culture systems are not fully synchronized, resulting in a combination of asexual and sexual stages present at the same time. Such a reality makes partial inhibitor interpretation difficult. Certainly, it is not clear if a drug needs to be active against both asexual and sexual stages of the parasite or if activity against only one of them is sufficient.

A way to circumvent this issue would be to use *C. parvum* transgenic strains (*Vinayak* et al.*, 2015*) that allow such elucidation of the life cycle of *C. parvum* (*Tandel* et al.*, 2019*). For the first time, the authors were able to distinguish the switch from asexual to sexual reproduction following three generations of meronts, and eventually, sexual stages developing directly from type I meronts rather than the previous postulate which indicated that sexual stages were derived from type II meronts. The mechanisms that control the switch from asexual to sexual stages are not yet understood. Targeting a specific phase of the *Cryptosporidium* life cycle seems to be a promising strategy for developing effective treatments, specifically to determine the effective treatment window for anti-*Cryptosporidium* compounds. Another advantage of transgenic strains is to enable studying the phenomenon of the parasite's resistance to drugs (*Hasan* et al.*, 2021*) or studying an essential gene function (*Choudhary* et al.*, 2020*). However, some limitations and questions have been pointed out, such as the technical challenge that obtaining transgenic parasites presents. Furthermore, the lingering question of whether transgene insertion may affect the parasite's biology or drug susceptibility remains. Can we translate these results to a native wild-type strain? A study on the marine natural product tartrolon E **(**TrtE) showed significant efficacy against *C. parvum in vitro* and *in vivo* in neonatal mice (*Bone Relat* et al.*, 2022*), investigating the specific life cycle phases and processes that TrtE targets. The authors demonstrated that between Nluc and WT *C. parvum* strains, there was no difference in their susceptibility to this compound, demonstrating that transgenic parasites can be used to explore the effects of TrtE on *C. parvum* with the same efficiency as with WT strains (*Cotto-Rosario* et al.*, 2022*).

Thus, the investigation of a drug activity at the sexual/asexual life stage remains technically limited. On the other hand, the identification of DMC1 protein expression coinciding with the appearance of the sexual stage and the selection of the DMC1 antibody by Jumani and colleagues (*Jumani* et al.*, 2019*) will potentially pave the way to the creation of a tool dedicated to the study of sexual development of *Cryptosporidium* in a High Throughput Screening assay.

It is noticeable that none of these cell culture improvements investigate the detection process in a context requiring the arsenal of analytical tools to be reinforced. This is because analytical processes supporting screening, irrespective of their advantages associated with rapid screening, still exhibit many disadvantages.

The high content imaging method supports the development of *C. parvum* and *C. hominis*. This technique allows readout at the same time for both host cells and *Cryptosporidium* growth. Moreover, the use of a 1536- well format is particularly interesting as it allows a high number of tests to be carried out at the same time. But the frequent requirement of fixation and staining steps make this a cumbersome process especially when a kinetic question is investigated. The requirement of special imaging equipment is also impeding the implementation of this method in all labs.

Considering the cytopathic effect method, inhibitors were tested against *C. parvum* and *C. hominis.* This technique is very simple and only requires a commercially available reagent to be added 24h post infection. But despite this cheap method allowing at the same time the selection of non-cytotoxic compounds and inhibitors, the infection step often requires large amounts of parasite. What's more, if the direct parasitic activity needs to be confirmed, this must be done in a secondary assay.

The quantitative real time PCR reverse transcription polymerase chain reaction (qRT-PCR) was used to study *C. parvum* growth inhibition. This method was of particular interest because it reduced the risk of errors due to dead parasites by the use of the whole well for lysate. Moreover, it can assess host cell quantification at the same time. For all that, this strategy exhibits several disadvantages. The use of 96-well plates limits its use as a high throughput method, although the recent use of 384-well plates could solve this question if laboratories invest in suitable thermocyclers. This requirement of specialized instrumentation associated with the need for high-cost reagents, represents a major limitation as well.

Finally, the luciferase-expressing reporter strain of *C. parvum* was presented as a simple method using a commercially available reagent leading to the production of a robust stable and quick luminescence. However, this genetically modified strain has restricted access.

As far as all these assays are concerned, they remain invasive as they require harvesting cells in order to label, to observe or to analyze genetic markers.

Taking all this into account, the development of analytical tools will require investigating methods at the interface of disciplines such as Biology and Physics. Therefore, cross-functional projects must be deployed using biophysical properties such as the electrical response of infected cells. For instance, a new strategy for HTS against *Cryptosporidium* could be derived from impedance spectroscopy. This method based on interdigitated planar microelectrodes exhibited highly interesting responses (*Dibao-Dina* et al.*, 2015*). When applying alternating current with a range of frequencies between electrodes, the authors demonstrated that infected cells exhibited a specific impedimetric signature directly linked to parasite viability. Considering this, adding new compounds into the growth medium for their parasite inhibitory effect would be very easy to do. Moreover, this method will not require any additional reagent. Impedance spectroscopy has potential advantages due to its sensitivity, non-invasiveness, and ability to provide real-time information automatically. Each dynamic change in the electrical properties of *Cryptosporidium* is tracked over time, which can provide kinetic data on the molecule's effect on parasites. However, the limitations of impedance spectroscopy lie not only in the ownership of the specialized equipment required, which can limit its accessibility in some laboratories, but also in the conception of a 96-well device for HTS. Developing this standard format will facilitate its accessibility and reduce the cost of acquisition. Previous results (internal data) showed its applicability as real-time monitoring of early parasitic stage development with alternating zoite and intracellular forms, observed respectively at peaks and local minima in the impedimetric signal. Also, thanks to differences in the magnitude of the impedimetric response, it can be used as an infectivity sensor which responds faster (under 12 h post-infection) than current methods (*Dibao-Dina* et al.*, 2015*). This method allows meaningful sample times to be defined, which would not have been visualized if the experiment was stopped at a fixed time (24h, 48h or 72 h). This is an opportunity to specifically target the window of action of anti- *Cryptosporidium* molecules.

## 6 Conclusion

The search for an efficient vaccine against *Cryptosporidium* is an ongoing process making significant progress, but which remains a preventive approach mostly applied to animal health (*Timmermans* et al.*, 2024*). As a result, therapeutic approaches remain important especially for human health. In this review we have presented the various ways screening for active molecules can be carried out and which pathways are currently targeted. It is clear that the diversity of experimental protocols carried out (cell type, animal, detection methods …) makes it difficult to compare the various activities and mechanisms. It would therefore be of benefit to the whole community to move towards some form of standardization. Moreover, it would be interesting to check the hits against various strains of *Cryptosporidium* to ensure the real-life efficacy of these molecules when they encounter a diversity of strains and, most importantly, when they come up against *C. hominis*.

## Funding sources

This project has received funding from the 10.13039/100013276Interreg 2 Seas Programme 2014–2020 co-funded by the 10.13039/501100008530European Regional Development Fund under subsidy contract No 2S05-043.

## CRediT authorship contribution statement

**Anne-Charlotte Lenière:** Writing – original draft, Formal analysis, Conceptualization. **Alexis Vlandas:** Writing – original draft, Supervision. **Jérôme Follet:** Writing – original draft, Supervision, Funding acquisition, Conceptualization.

## Declaration of competing interest

None.
